# Two new species of the genus *Anufrievia* Dworakowska from China (Hemiptera: Cicadellidae: Typhlocybinae)

**DOI:** 10.3897/BDJ.9.e68043

**Published:** 2021-06-03

**Authors:** Weiwen Tan, Jia Jiang, Yuehua Song

**Affiliations:** 1 School of Karst Science, Guizhou Normal University / State Key Laboratory Cultivation Base for Guizhou Karst Mountain Ecology Environment of China, Guiyang, 550001, China School of Karst Science, Guizhou Normal University / State Key Laboratory Cultivation Base for Guizhou Karst Mountain Ecology Environment of China Guiyang, 550001 China

**Keywords:** Homoptera, Auchenorrhyncha, Erythroneurini, taxonomy, leafhopper

## Abstract

**Background:**

The leafhopper genus *Anufrievia* Dworakowska, 1970 includes 33 species and is widely distributed in China, Korea, South Korea, Japan, Nepal, India, Thailand and Vietnam.

**New information:**

Two new species found at Bijie City and Shibing County, Guizhou Province, China are described and illustrated, *A. crispata* **sp. nov.** and *A. confluensa* **sp. nov.** A key to distinguish the Chinese species of the genus is given.

## Introduction

The leafhopper genus *Anufrievia* Dworakowska, 1970 belongs to the tribe Erythroneurini of Typhlocybinae, with *Anufrievia rolikae* Dworakowska, 1970 as its type species (Dworakowska 1970). The genus previously contained 33 species, including 28 species in China (Cao et al. 2018). In this paper, two new species from Guizhou Province, China are described and illustrated and a key to distinguish the Chinese species of the genus is provided.

The characteristics of *Anufrievia* genus are as follows. Body yellow or white, often with brown markings or diffuse patterns. Head slightly narrower than pronotum. Length of crown distinctly shorter than inter ocular width. Body length 2.0–4.0 mm (including wing). Vertex anterior margin with minute paired black spots. Male anteclypeus narrow and flat, greyish, brown or black. Pronotum pale, scutellum with dark lateral triangles. Forewing with 4^th^ apical cell small, not reaching apex of forewing, 2^nd^ apical cell nearly rectangular and 1^st^ apical cell broad. Hind wing venation follows typical schemes for Erythroneurini taxa. Abdominal apodemes small and narrow, extended dorsomesad.

Male pygofer lobe with hind margin sleeked or truncated slightly, basal lateral angle usually with macrosetae, sometimes absent and scattered a few fine setae in outer lateral surface. Pygofer dorsal appendage articulated to pygofer lobe with ventral appendage absent. Subgenital plate with some macrosetae in mid-ventral part, broad basally and sometimes terminal half tapering abruptly; row of stout setae along upper margin from sub-base to apex. Apex of style pointed, bifid, foot-shaped or otherwise modified. Aedeagus with dorsal apodeme well developed; aedeagal shaft tubular; gonopore sub-basal to subapical on ventral surface. Connective lateral arms long, Y- or V-shaped.

## Materials and methods

All specimens in this study were collected by the sweeping-net method. Morphological terminology used follows Dietrich (2005) and Dworakowska (1993). An Olympus SZX16 dissecting microscope was used for observing and an Olympus BX53 stereomicroscope for drawing. A KEYENCE VHX-5000 digital microscope was used for taking habitus photos. Body measurements are from the apex of the vertex to the tip of the forewing. All specimens examined were deposited in the collection of the School of Karst Science, Guizhou Normal University, China (GZNU).

## Taxon treatments

### Anufrievia crispata sp. n.

AFC89331-A7C4-5004-B3A4-3670E2076383

6C5F6BDB-CDAB-4407-857F-72CF40893FF7

#### Materials

- **Type status:** Holotype. **Occurrence:** individualCount: 1; sex: male; lifeStage: adult; **Taxon:** scientificName: *Anufrievia crispata*; order: Hemiptera; family: Cicadellidae; genus: Anufrievia; specificEpithet: *crispata*; **Location:** country: China; stateProvince: Guizhou; locality: Bijie City, Qixinguan District, Salaxi Town; locationRemarks: label transliteration: "Guizhou, Bijie, 24. 10. 2019, coll. Zhouwei Yuan and Xiao Yang"; **Record Level:** collectionCode: Insects; basisOfRecord: PreservedSpecimen
- **Type status:** Paratype. **Occurrence:** individualCount: 4; sex: male; lifeStage: adult; **Taxon:** scientificName: *Anufrievia crispata*; order: Hemiptera; family: Cicadellidae; genus: Anufrievia; specificEpithet: *crispata*; **Location:** country: China; stateProvince: Guizhou; locality: Bijie City, Qixinguan District, Salaxi Town; locationRemarks: label transliteration: "Guizhou, Bijie, 24.10. 2019, coll. Zhouwei Yuan and Xiao Yang"; **Record Level:** collectionCode: Insects; basisOfRecord: PreservedSpecimen

#### Description

**Figure 1.**
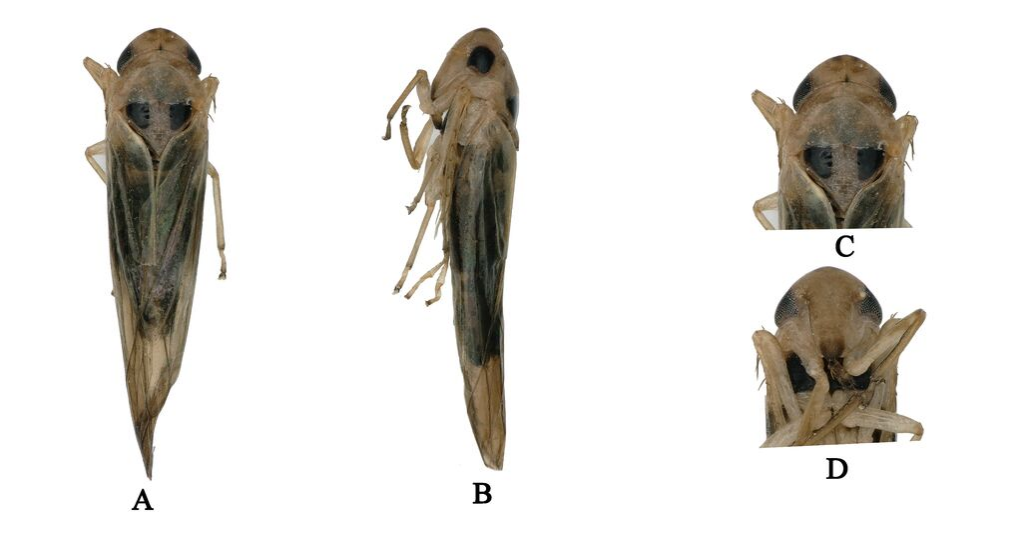
*Anufrievia crispata* sp. nov. **A.** Habitus, dorsal view; **B.** Habitus, lateral view; **C.** Head and thorax, dorsal view; **D.** Face.

**Figure 2.**
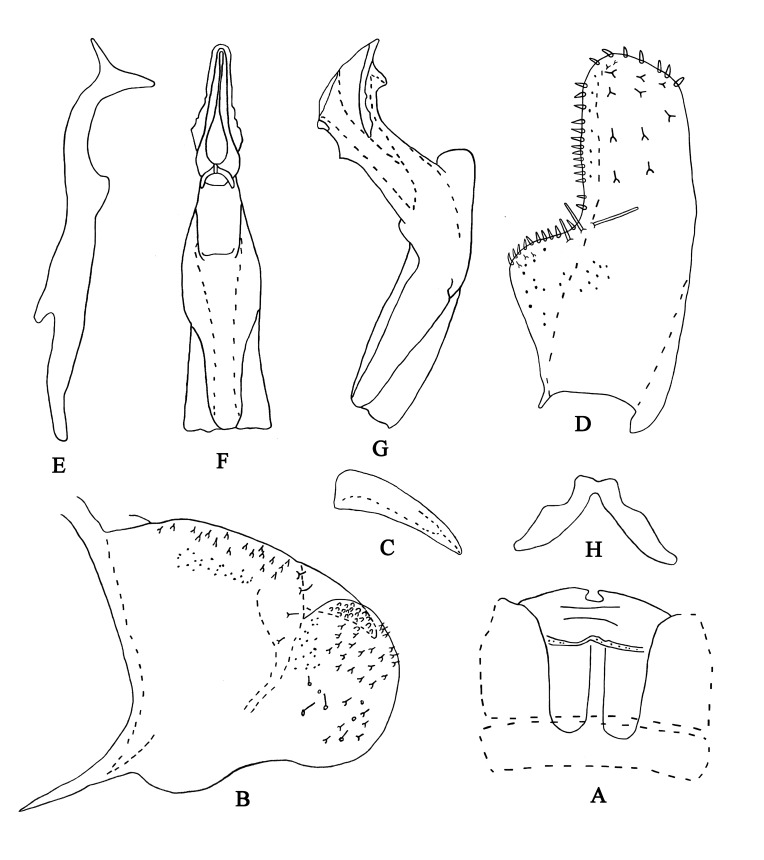
*Anufrievia crispata* sp. nov. **A.** Abdominal apodemes; **B.** Pygofer lobe; **C.** Pygofer dorsal appendage, lateral view; **D.** Subgenital plate; **E.** Style **F.** Aedeagus, ventral view; **G.** Aedeagus, ventrolateral view; **H.** Connective.

Body brownish-black. Head brownish-yellow, with pair of small dark brown apical spots (Fig. 1A and C). Eyes black. Face brownish-yellow, frontoclypeus brownish and anteclypeus centrally brown with black lateral margins (Fig. 1B and D). Pronotum light brownish (Fig. 1A and C). Scutellum brownish-yellow, with black basal triangles (Fig. 1A and C). Forewing beige (Fig. 1A and B). Abdominal apodemes broad, extended to 4^th^ sternite (Fig. 2A). Male length 3.7–4.0 mm (including wing).

#### Diagnosis

**Male genitalia.** Pygofer lobe with numerous microsetae distributed densely along dorsal and near posterior margin, few fine setae scattered on lateral surface (Fig. 2B). Pygofer dorsal appendage broadened at base, tapering towards apex (Fig. 2C). Subgenital plate robust, with three macrosetae near mid-length on lateral surface, several peg-like setae distributed from sub-base to apex; several microsetae scattered on apical portion (Fig. 2D). Style with two points apically; pre-apical lobe prominent (Fig. 2E). Aedeagal shaft curved dorsally, with serrated marginal lamellae on shaft; pair of small processes curved mesally gonopore; subapical on ventral surface (Fig. 2F and G). Connective V-shaped, slender (Fig. 2H).

#### Etymology

The new species is named from the Latin word “*crispatus*”, referring to the serrated marginal lamellae on both sides of shaft apex dorsad (Fig. 2F and G).

#### Taxon discussion

This species can be distinguished from other species in this genus by the unique characters of the aedeagus: the aedeagal shaft with serrated marginal lamellae on both sides of apex, pair of small curved processes subapically; short dorsal apodeme and long prearium.

### Anufrievia confluensa sp. n.

2693AF42-57EA-5D51-BDB4-AC52ABBC25FE

67AE0DA0-127A-4D2B-9B54-FD0CD9EC78C4

#### Materials

- **Type status:** Holotype. **Occurrence:** individualCount: 1; sex: male; lifeStage: adult; **Taxon:** scientificName: *Anufrievia confluensa*; genus: Anufrievia; specificEpithet: *confluensa*; **Location:** country: China; stateProvince: Guizhou; county: Shibing; locationRemarks: label transliteration: "Guizhou, Shibing, 24.7.2019, coll. Zhouwei Yuan and Xiao Yang”; **Record Level:** collectionCode: Insects; basisOfRecord: PreservedSpecimen

#### Description

**Figure 3.**
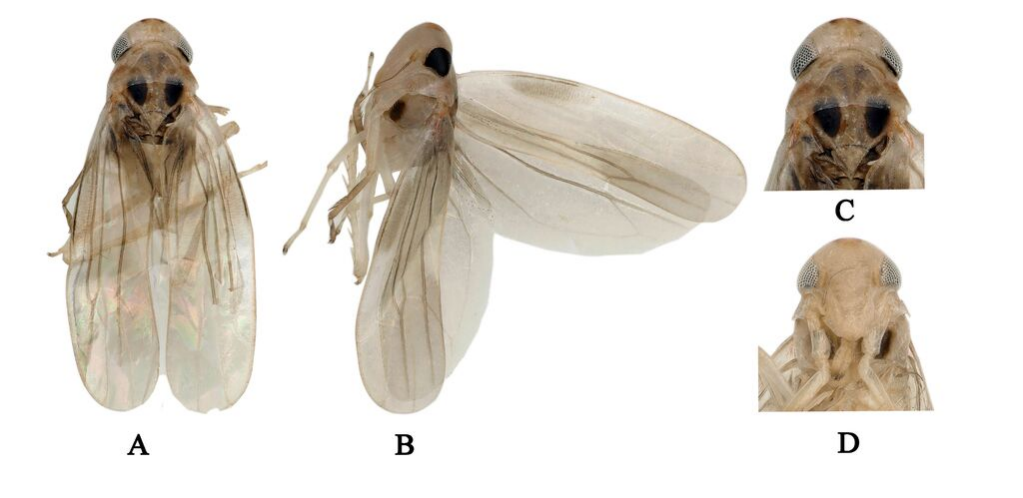
*Anufrievia confluensa* sp. nov. **A.** Habitus, dorsal view; **B.** Habitus, lateral view; **C.** Head and thorax, dorsal view; **D.** Face.

**Figure 4.**
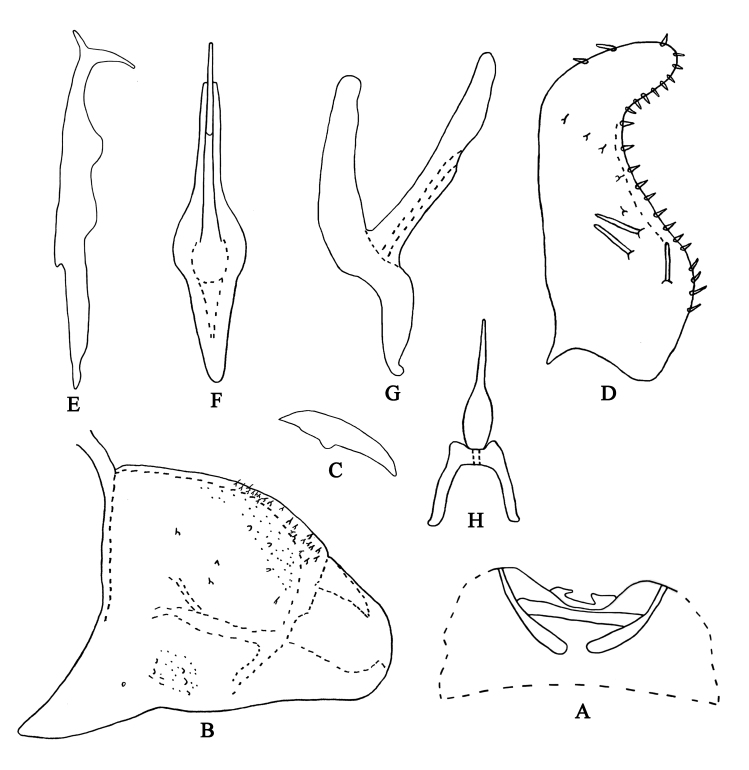
***Anufrievia confluensa* sp. nov. A.** Abdominal apodemes; **B.** Pygofer lobe; **C.** Pygofer dorsal appendage, lateral view; **D.** Subgenital plate; **E.** Style; **F.** Aedeagus, ventral view; **G.** Aedeagus, lateral view; **H.** Connective.

Male length 2.8 mm (including wing). Body yellowish. Vertex brownish-yellow, with pair of small dark brown apical spots (Fig. 3A and C). Eyes grey. Face pale milky yellow, anteclypeus and frontoclypeus light brownish (Fig. 3B and D). Pronotum and scutellum brownish-yellow and anterior margin of scutellum with black triangles (Fig. 3A and C). Forewing beige (Fig. 3A and B). Abdominal apodemes very short, not extended to 3^rd^ sternite (Fig. 4A).

#### Diagnosis

**Male genitalia.** Pygofer lobe broad, with dense microsetae near dorso-caudal margin and several peg-like setae on outer surface (Fig. 4B). Pygofer dorsal appendage broadened at base, tapering towards apex (Fig. 4C). Subgenital plate slightly concave near middle area, with three macrosetae on lateral margin, row of short stout setae along upper margin from sub-base to apex (Fig. 4D). Style with two points at apex; pre-apical lobe small (Fig. 4E). Aedeagal shaft straight and flat in lateral view, but long and slim in ventral view; gonopore arising from ventral surface, reaching two thirds of aedeagal shaft; dorsal apodeme well developed (Fig. 4F and G). Connective Y-shaped, two arms slender, central lobe absent (Fig. 4H).

#### Etymology

The new species is named from the Latin word “*confluensus*”, referring to the connective stem fused with a long process (Fig. 4H).

#### Taxon discussion

This species is similar to *A. akazu* (Matsumura 1932), but can be recognised by the subapical ventral surface without paired short processes and pygofer appendage not bifurcate at apex.

## Identification Keys

### Key to males of *Anufrievia* from China (modified from Cao et al. 2018)

**Table:** 

1	Pygofer dorsal appendage not bifurcate at apex	2
–	Pygofer dorsal appendage bifurcate at apex	11
2	Aedeagus with large dorsal apodeme	3
–	Aedeagus with small dorsal apodeme	*A. crispata* sp. nov
3	Pre-atrial process not reaching gonopore	4
–	Pre-atrial process reaching or surpassing gonopore	8
4	Style without distinct apical and subapical teeth	5
–	Style with distinct apical and subapical teeth	6
5	Style with apex slim (Fig. 5T)	*A. symmetrica* Cao & Zhang
–	Style with apex triangular (Fig. 5U)	*A. triangulata* Cao & Zhang
6	Pre-atrial process almost rectangular in ventral view, apex broad (Fig. 5O)	*A. quadrata* Cao & Zhang
–	Pre-atrial process narrowing apically, apex pointed	7
7	Style with subapical tooth equal in length to apical tooth (Fig. 5A)	*A. adaucta* Cao & Zhang
–	Style with subapical tooth shorter than apical tooth (Fig. 5P)	*A. sphenoides* Yang & Zhang
8	Aedeagal shaft with pair of apical processes	9
–	Aedeagal shaft without any apical process	*A. confluensa* **sp. nov**
9	Aedeagal apical processes arched medially in ventral view (Fig. 5B)	*A. arcuata* Yang & Zhang
–	Aedeagal apical processes slightly curved in ventral view	10
10	Aedeagal shaft with base slim, slightly wider than apex (Fig. 5X)	*A. zelta* Dworakowska
–	Aedeagal shaft with base broad, much wider than apex	11
11	Aedeagal shaft constricted sub-basally (Fig. 5J)	*A. jinghongensis* Cao & Zhang
–	Aedeagal shaft not constricted sub-basally	12
12	Style with apical tooth extremely small, aedeagal shaft straight (Fig. 5R)	*A. subdentata* Yang & Zhang
–	Style with apical tooth relatively long, aedeagal shaft curved dorsad	*A. ciconia* Dworakowska
13	Aedeagal shaft with processes near middle (Fig. 5V)	*A. triprocessa* Yang & Zhang
–	Aedeagal shaft without process near middle	14
14	Apex of style serrated at middle	15
–	Apex of style smooth at middle	18
15	Upper tooth of pygofer dorsal appendage much shorter than lower one (Fig. 5D)	*A. bauhinicola* Dworakowska & Viraktamath
–	Upper tooth of pygofer dorsal appendage subequal to or longer than lower one	16
16	Upper tooth of pygofer dorsal appendage longer than lower one (Fig. 5F)	*A. expansa* Cao & Zhang
–	Upper tooth of pygofer dorsal appendage almost as long as lower one	17
17	Apex of pre-atrial process rounded, with one side serrated (Fig. 5M)	*A. plana* Yang & Zhang
–	Apex of pre-atrial process truncate, with both sides smooth (Fig. 5E)	*A. curva* Yang & Zhang
18	Ventral margin of aedeagal shaft protruded subapically in lateral view	19
–	Ventral margin of aedeagal shaft straight subapically, in lateral view	21
19	Apical tooth of style almost equal to subapical tooth (Fig. 5K)	*A. liubanus* Yang & Zhang
–	Apical tooth of style greatly shorter than subapical tooth	20
20	Aedeagal shaft processes relatively long, gonopore central (Fig. 5L)	*A. parisakazu* Cao & Zhang
–	Aedeagal shaft processes relatively short, gonopore subapical	*A. akazu* Matsumura
21	Apex of pre-atrial process serrated laterally (Fig. 5I)	*A. fusina* Yang & Zhang
–	Apex of pre-atrial process smooth	22
22	Pre-atrial process rudimentary, as long as 1/5 of aedeagal shaft (Fig. 5C)	*A. badjawae* Dworakowska
–	Pre-atrial process much longer than 1/5 of aedeagal shaft	23
23	Aedeagal shaft curved dorsad (Fig. 5G)	*A. falcata* Yang & Zhang
–	Aedeagal shaft straight	24
24	Apex of style slender (Fig. 5N)	*A. qinlingensis* Yang & Zhang
–	Apex of style foot-like	25
25	Aedeagal shaft with processes arising from subapex	26
–	Aedeagal shaft with processes arising from apex	27
26	Apex of aedeagal shaft expanded (Fig. 5H)	*A. forcipiformis* Yang & Zhang
–	Apex of aedeagal shaft narrow (Fig. 5Q)	*A. subapicifixa* Yang & Zhang
27	Aedeagal shaft processes bent at right angle in ventral view	*A. rolikae* Dworakowska
–	Aedeagal shaft processes straight or slightly curved in ventral view	29
28	Style without distinct apical and subapical teeth (Fig. 5S)	*A. sufflata* Yang & Zhang
–	Style with distinct apical and subapical teeth	29
29	Gonopore subapical (Fig. 5W)	*A. wolongensis* Yang & Zhang
–	Gonopore central	*A. maculosa* Dworakowska

## Supplementary Material

XML Treatment for Anufrievia crispata

XML Treatment for Anufrievia confluensa

## Figures and Tables

**Figure 5.**
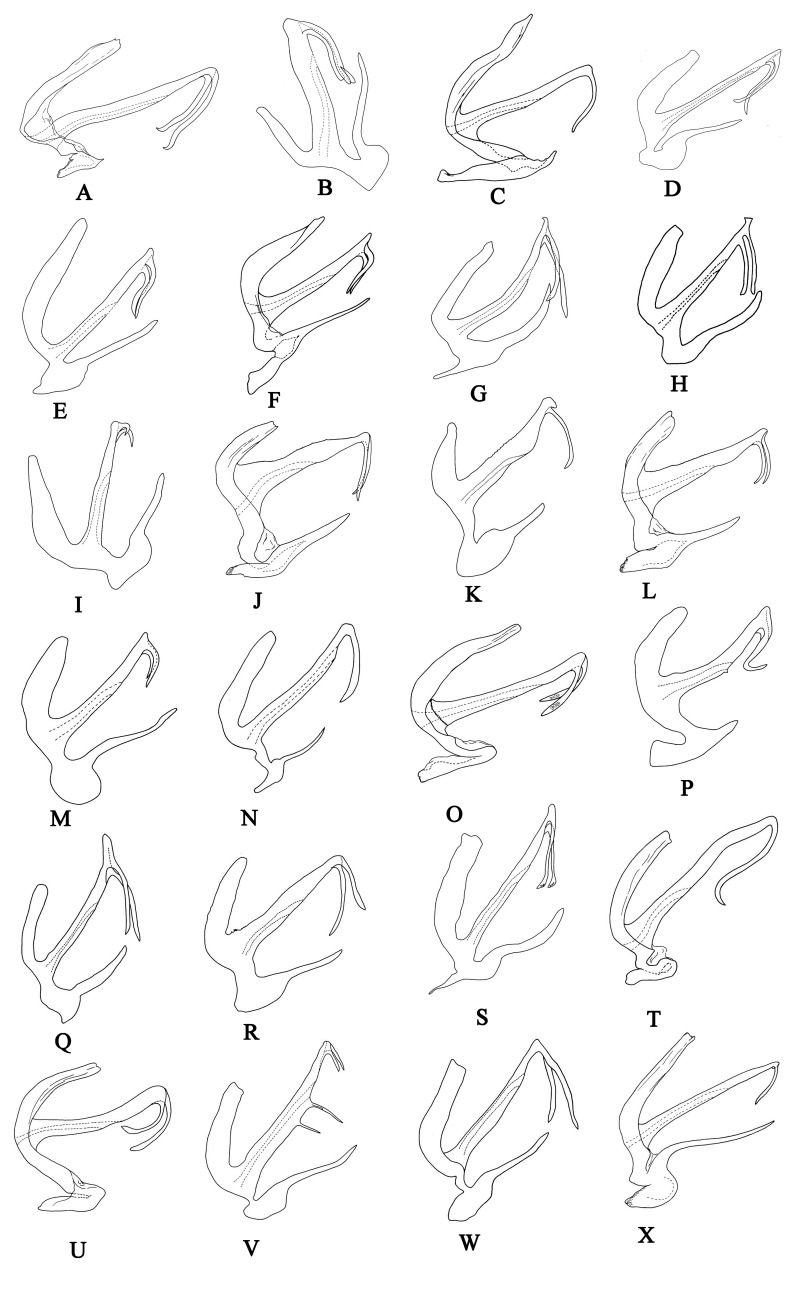
Aedeagus of Anufrievia spp, lateral view. **A.** *A. adaucta* Cao & Zhang, 2018; **B.** *A. arcuata* Yang & Zhang, 2018; **C.** *Anufrievia badjawae* Dworakowska, 1976; **D.** *Anufrievia bauhinicola* Dworakowska & Viraktamath, 1978; **E.** *A. curva* Yang & Zhang, 2018; **F.** *A. expansa* Cao & Zhang, 2018; **G.** *A. falcata* Yang & Zhang, 2018; **H.** *A. forcipiformis* Yang & Zhang, 2018; **I.** *A. fusina* Yang & Zhang, 2018; **J.** *A. jinghongensis* Cao & Zhang, 2018; **K.** *A. liubanus* Yang & Zhang, 2018; **L.** *A. parisakazu* Cao & Zhang, 2018; **M.** *A. plana* Yang & Zhang, 2018; **N.** *A. qinlingensis* Yang & Zhang, 2018; **O.** *A. quadrata* Cao & Zhang, 2018; **P.** *A. sphenoides* Yang & Zhang, 2018; **Q.** *A. subapicifixa* Yang & Zhang, 2018; **R.** *A. subdentata* Yang & Zhang, 2018; **S.** *A. sufflata* Yang & Zhang, 2018; **T.** *A. symmetrica* Cao & Zhang, 2018; **U.** *A. triangulata* Cao & Zhang, 2018; **V.** *A. triprocessa* Yang & Zhang, 2018; **W.** *A. wolongensis* Yang & Zhang, 2018; **X.** *Anufrievia zelta* Dworakowska, 1977.
